# Palmitoylethanolamide (PEA) regulates cell cycle progression and promotes an anti‐inflammatory transcriptomic signature in C2C12 skeletal muscle cells

**DOI:** 10.14814/phy2.70780

**Published:** 2026-02-15

**Authors:** Paige L. Cole, Scott H. Gillham, Mark R. Viggars, Graeme L. Close, Daniel J. Owens

**Affiliations:** ^1^ Research Institute of Sport and Exercise Science (RISES) Liverpool John Moores University Liverpool UK; ^2^ Department of Physiology and Aging University of Florida Gainesville Florida USA

**Keywords:** immunomodulation, myogenesis, N‐acylethanolamines

## Abstract

Palmitoylethanolamide (PEA) is an endogenous lipid mediator with immunomodulatory actions, yet its effects in skeletal muscle remain poorly defined. We examined whether PEA influences myogenesis and profiled the acute transcriptomic response of differentiated C2C12 myotubes to 10 μM PEA. PEA decreased myotube number (90.3 ± 10.6 vs. 112.6 ± 10.1 control) while increasing nuclear fusion index (37.8 ± 5.7% vs. 30.7 ± 3.2%); myotube area was unchanged. In myoblasts, 24 h PEA increased G_0_/G_1_ (48.2 ± 1.2% vs. 42.3 ± 1.9%) and reduced S‐phase (21.7 ± 1.2% vs. 25.5 ± 1.2%), consistent with G_1_ arrest. RNA sequencing identified 1952 differentially expressed genes enriched for cytokine–receptor interactions and inflammatory signaling. PEA downregulated NF‐κB target cytokines while upregulating interferon‐related and chemokine genes, indicating an anti‐inflammatory/immune‐priming profile. N‐acylethanolamine acid amidase was highly expressed and induced, whereas fatty acid amide hydrolase remained low and unchanged, suggesting muscle‐specific reliance on NAAA metabolism. These data show that PEA biases skeletal muscle toward a less proliferative but more fused and inflammation‐resolving phenotype, with transcriptional reprogramming of immune pathways and preferential NAAA engagement. These findings motivate in vivo studies to test whether such actions benefit muscle regeneration, adaptation, or anti‐atrophy interventions.

## 1 INTRODUCTION

Palmitoylethanolamide (PEA) is an endogenous fatty acid amide with well‐documented anti‐inflammatory, analgesic, and neuroprotective properties (Petrosino & Di Marzo, 2017; Skaper et al., 2015). Endogenous PEA is biosynthesized from the membrane phospholipid N‐palmitoyl‐phosphatidylethanolamine (NAPE), most prominently via hydrolysis by N‐acyl phosphatidylethanolamine‐specific phospholipase D (NAPE‐PLD) in neuronal, glial, and immune cells (Iannotti et al., 2016). Following synthesis, PEA is degraded by fatty acid amide hydrolase (FAAH) or N‐acylethanolamine‐hydrolysing acid amidase (NAAA). PEA acts through multiple signaling pathways, including direct activation of GPR55 and PPAR‐α, and indirect modulation of TRPV1 channels and CB2 receptors via PPAR‐α‐dependent mechanisms (Di Cesare Mannelli et al., 2013). PEA may also enhance endocannabinoid signaling by elevating anandamide (AEA) and 2‐arachidonoylglycerol (2‐AG), contributing to the so‐called “entourage effect” (De Petrocellis et al., 2001). While these mechanisms are well described in neural and immune cells, their presence and relevance in other tissues remain unclear.

Through activation of these signaling networks, PEA has been shown to modulate inflammatory and nociceptive pathways, making it a promising candidate for conditions characterized by pain, inflammation, and neurodegeneration. Given the close interplay between inflammation and skeletal muscle function, PEA has attracted attention for its potential role in muscle health, particularly in the context of pain, inflammation, degeneration, and exercise‐induced muscle damage (EIMD). Preclinical models demonstrate that PEA supplementation accelerates muscle repair by reducing pro‐inflammatory cytokine expression and promoting tissue regeneration (Lo Verme et al., 2005). PEA's interaction with PPAR‐α has been identified as a central mechanism driving these anti‐inflammatory and pro‐resolving effects (D'Agostino et al., 2012). Micronized PEA formulations have further been shown to reduce nitrosative stress in peripheral tissues, such as carrageenan‐induced paw oedema, by decreasing nitrotyrosine formation and myeloperoxidase activity (Impellizzeri et al., 2014). To our knowledge, similar antioxidant effects of PEA have not yet been investigated in skeletal muscle tissue, where oxidative stress also plays a key role in damage, recovery and adaptation. Clinical investigations provide further support for PEA's role in pain management and muscle health. A meta‐analysis by Paladini et al. (2016) highlighted significant pain relief in patients with chronic pain conditions, including musculoskeletal disorders, with PEA supplementation improving quality of life and functional outcomes, while an exploratory study in elderly individuals with sarcopenia suggested improvements in muscle strength and performance following PEA supplementation (Gatti et al., 2012).

Beyond its anti‐inflammatory and analgesic actions, PEA may also influence energy metabolism. Through PPAR‐α activation, PEA regulates fatty acid oxidation and mitochondrial function in metabolically active tissues (Lo Verme et al., 2005). In non‐muscle models, PEA has been associated with improved mitochondrial respiration and reduced oxidative damage. For example, in diet‐induced obese mice, oral PEA enhanced hepatic mitochondrial oxidative capacity, improved energy efficiency, and reduced lipid accumulation via PPAR‐α and AMPK pathways (Annunziata et al., 2020). Ultramicronized PEA has also been shown to preserve respiratory complex I and FoF1 ATPase activity under inflammatory stress in neural models (Bellanti et al., 2022). Whether these PPAR‐α‐mediated effects of PEA extend to skeletal muscle, a tissue with high metabolic and mitochondrial demands, remains unknown. In addition, PEA's neuroprotective effects, including modulation of mast cell activity and neuroinflammation (Gabrielsson et al., 2016), could indirectly benefit muscle function by alleviating neuropathic pain often associated with muscle disorders. Nonetheless, the direct impact of PEA on mitochondrial pathways and energy metabolism in skeletal muscle has not yet been elucidated.

Taken together, the direct effects of PEA on skeletal muscle remain poorly understood. Specifically, little is known about its role in skeletal muscle myogenesis or the transcriptional response of muscle cells to PEA exposure. These fundamental insights could improve the quality of PEA research in muscle and pave the way to future work. Therefore, the specific aims of this study were to (i) investigate if PEA impacts skeletal muscle myogenesis and (ii) explore the acute transcriptional response of skeletal myotubes following PEA treatment, thereby providing new insights into the molecular mechanisms underlying PEA's effects on muscle tissue.

## 2 MATERIALS AND METHODS

### 2.1 Chemicals, reagents, and plasticware

Palmitoylethanolamide (PEA) was purchased from Sigma‐Merck (Cat No. P0359) and reconstituted in DMSO as per manufacturer guidelines. Reconstituted PEA was stored at −20°C and used within 3 weeks of reconstitution. When required, PEA stock solution was diluted further in culture media for the experiments outlined herein.

Dulbecco's modified eagle medium (DMEM: Gibco 11,995,073), fetal bovine serum (FBS: Gibco Cat No. A5256701), new‐born calf serum (NBCS: Gibco Cat No. 26010074), penicillin–streptomycin (pen‐strep: Gibco Cat No. 15070063), trypsin–EDTA (Gibco Cat No. 25200056), horse serum (HS: Gibco Cat No. 26050088), H_2_O_2_ (Thermo Scientific Cat No. L14000.AP), propidium iodide (Invitrogen Cat No. P1304MP), and MTT (3‐(4,5‐Dimethylthiazol‐2‐yl)‐2,5‐Diphenyltetrazolium Bromide; Invitrogen Cat No. M6494) were purchased from Thermo Fisher Scientific (Oxford, UK). Phosphate buffered saline (PBS: Sigma‐Aldrich Cat No. P4417) tablets, DMSO (Sigma‐Aldrich Cat No. 472301), and gelatin from porcine skin (Sigma‐Aldrich Cat No. G9136) were purchased from Sigma Aldrich (Gillingham, UK). Nunc T75 culture flasks and 6 well culture plates (Nunc Cat No. 150239) were purchased from Thermo Fisher Scientific.

C2C12 myoblasts were purchased from ATCC (LGC Standards, Middlesex, UK). All experiments detailed in this manuscript were performed on C2C12s between passage 5 and 10. For the isolation of high‐quality RNA, RNeasy isolation kits were purchased from Qiagen (Cat No. 74134. Qiagen Ltd. Manchester, UK).

### 2.2 Cell culture

C2C12 murine myoblasts were cultured on gelatin (0.2%) coated 6‐well culture plates in humidified 5% CO_2_ at 37°C in growth media comprising DMEM, 10% FBS, 10% NBCS and 1% of a pen‐strep solution. Upon reaching ~80% confluence, monolayers were washed twice with pre‐warmed PBS and switched to low serum differentiation media (DM), DMEM, 2% HS and 1% pen‐strep. Every 48 h thereafter, DM was removed from monolayers via aspiration and replaced with fresh media. Cells were terminally differentiated by Day 8 of low serum DM exposure.

### 2.3 Dose tolerability experiments

To ascertain an appropriate dose that would maximize the signal but minimize toxicity to the terminally differentiated C2C12 myotubes used in this study, we relied on previous literature to develop our own dose tolerance experiments. Given the lack of research regarding the effects of PEA on the viability of C2C12 skeletal muscle cells, we determined our dosage selections on those used within general cell culture studies across multiple studies. Previous studies have examined the effects of 0.1, 0.5, 1, and 10 μM PEA on RBL‐2H3 cells, a basophilic leukemia cell line, and discovered no detrimental effects on viability and cytotoxicity at the maximum concentration of 10 μM (Petrosino et al., 2019). Additionally, another study identified 100 μM as the optimal dose for reducing the effects of lipopolysaccharide (LPS) on N9 microglia cells (D'Aloia et al., 2021). While these studies were conducted on different cell lines, considering these findings collectively, we investigated 1, 10, and 100 μM of PEA. We utilized a metabolic activity assay and propidium iodide exclusion assay to determine the cytotoxicity of PEA.

#### 2.3.1 3‐(4,5‐dimethylthiazol‐2‐yl)‐2,5‐diphenyltetrazolium bromide (MTT) metabolic activity assay

NAD(P)H‐dependent cellular oxidoreductase enzymes may reflect the number of viable cells present in cell culture models (Stockert et al., 2018). These enzymes can reduce the tetrazolium dye MTT to its insoluble formazan, which has a purple color. The amount of formazan produced is directly proportional to the number of living cells present in the culture, however, decreases in cellular metabolic activity may occur before cell death and as such, the MTT assay was used in parallel to the propidium iodide assay described in the following section to determine the dose‐responsiveness of C2C12s to varying CBD concentrations.

C2C12 myoblasts were seeded at 6 × 10^4^ cells mL^−1^ in pregelatinized 6‐well plates in GM and cultured to 80% confluence. Thereafter, cells were induced to terminally differentiate as described under the cell culture section above. After 8 days of differentiation, existing DM was removed from monolayers. Following three PBS washes, monolayers were treated with either DM containing vehicle solution DMSO (CON) or DM + PEA at doses from 1 to 100 μM (Figure 2). Following 24 h treatment, MTT solution was added to each well at 10% of total well volume and incubated for 180 min. Thereafter, existing media was removed from monolayers before another short incubation of 6 min, which was completed with plate lids removed. Five hundred microlitres of DMSO was then added to each well, and plates were agitated on a plate rocker at 120 rpm for 2 min to ensure all cells had detached from the plate. Plates were then positioned into a Spark multi‐mode microplate reader (Tecan, Mannedorf, Switzerland) and measured at a wavelength of 570 nm to detect the change in absorbance.

#### 2.3.2 Cell viability assay (propidium iodide exclusion)

In a separate experiment, myoblasts were seeded at 6 × 10^4^ cells·mL^−1^ in pregelatinized 6‐well plates in GM and cultured to 80% confluence. Thereafter, cells were induced to terminally differentiate as described under the cell culture section above. On day 8 of differentiation, existing media was removed from monolayers, which were subsequently treated with either DM containing vehicle solution DMSO (CON) or DM + PEA at doses from 1 to 100 μM. Following 24 h treatment, existing media was pipetted into 1.5 mL Eppendorf tubes and monolayers were washed twice with prewarmed PBS. Thereafter, trypsin–EDTA 0.025% was added to each well and plates were incubated for 5 min (37°C, 5% CO_2_). The removed media was then placed back onto its corresponding well to allow serum to neutralize the activity of trypsin. This solution was then pipetted into a 1.5 mL Eppendorf and centrifuged for 5 min at 300×*g*. Following centrifugation, the existing media was aspirated from each Eppendorf, leaving a small pellet of cells. Two hundred microlitres of fresh DM was then added to each Eppendorf, and cell pellets were fully resuspended by pipetting to create a solution of cells and DM. Propidium Iodide (5 mg·mL^−1^ in ddH_2_O) was then added to each Eppendorf at a concentration of 1:100 and these were vortexed for 20 s before a 5‐min incubation (37°C, 5% CO_2_). PI fluorescence was then analyzed via flow cytometry using the FL‐3 channel on a BD AccuriTM C6 Plus Flow Cytometer (BD Biosciences, Berkshire, UK). First, negative and positive controls were established whereby PI‐free cells were analyzed for background fluorescence alongside cells treated with 50% *v/v* H_2_O_2_. PI‐free cells had no increase in fluorescence, while H_2_O_2_ increased fluorescence (Data not shown). The excitation/emission maximum of the dye is typically 493/636; upon binding to DNA, excitation/emission maxima increased to 535/617 nm.

### 2.4 Next generation RNA sequencing

#### 2.4.1 Cell treatments

After assessing the tolerability of C2C12 myotubes to different doses of PEA, we noted a significant decrease in metabolic activity and cell viability at 100 μM PEA, as indicated by the MTT and PI assays. Therefore, we selected 10 μM PEA for subsequent experiments. To determine the transcriptional response to 10 μM PEA, we terminally differentiated C2C12 myotubes as described above. On day 8 of differentiation, the differentiation medium (DM) was removed and replaced with DM containing either vehicle (DMSO) or PEA (10 μM). Myotubes were then cultured in the respective treatment for 24 h before being harvested for RNA extraction. Duplicate wells (technical replicates) were used for each treatment, and the experiment was repeated three times (experimental replicates), generating *n* = 6 samples per treatment.

#### 2.4.2 RNA isolation

Following a 24‐h treatment with PEA or vehicle control, terminally differentiated C2C12 monolayers were lysed with buffer RLT from the Qiagen RNeasy kit (Qiagen Ltd.: Cat No. 74134). RNA was then extracted from cell lysates using the RNeasy kit with proteinase K digestion, as per the manufacturer's guidelines. Eluted RNA was stored at −80°C until required for determination of total RNA and library processing.

#### 2.4.3 Determination of RNA quantity and quality

Total RNA was quantified using a Nanodrop 8000 and RNA quality was assessed using an Agilent® Bioanalyser (average RIN score = 7, 260/280 = 2.1, 260/230 = 1.7). RNA samples were then diluted to 20 ng μL^−1^ using RNase‐free water.

#### 2.4.4 RNA library preparation and sequencing

Libraries were constructed from 100 ng of total RNA with Poly‐A tail enrichment of mRNA using NEBNext® Ultra™ II RNA Library Prep Kit for Illumina® with Agencourt AMPureXP Sample Purification Beads (Cat No. E7775L. Beckman Coulter. Wycombe, UK) as per manufacturers guidelines, by Bart's and the London Genome Centre at Queen Mary, University of London. The resultant‐barcoded libraries were sequenced on an Illumina NextSeq 2000 using 2 × 50 bp paired‐end sequencing. An average of 25 million paired‐end reads was achieved per sample.

#### 2.4.5 RNA sequencing

FastQ files were imported to Partek® Flow® Genomic Analysis Software Partek Inc. (Missouri, USA) for pipeline processing. Pre‐alignment QA/QC was performed on all reads prior to read trimming (Reads with Phred score <20 removed). STAR alignment 4.4.1d was used to align trimmed reads to the *Mus musculus* genome, mm39. Aligned reads were then quantified to the Ensembl transcriptome annotation model v.100. Post alignment QC reports are provided in Table S1. Filtered raw counts were used for normalization and differential analysis with DESeq2 (Love et al., 2014) through Partek® Flow®. Gene transcripts were considered significantly different between groups when the *q*‐value <0.05. Volcano plots and annotation charts were generated in R studio (version 2023.06.1 + 524), Principal Component Analysis (PCA) plots were generated in Partek® Flow® and bar charts were constructed in GraphPad Prism version 10.0.0 for Windows (GraphPad Software, Boston, Massachusetts USA). To facilitate further discovery and reproducibility, all RNA‐seq data from this study are openly available via the Gene Expression Omnibus (GEO accession number GSE307376).

### 2.5 Immunofluorescence (IF) staining

C2C12 myoblasts were seeded at 6 × 10^4^ cells mL^−1^ in pregelatinized 6‐well plates in GM and cultured to 80% confluence. Existing GM was then aspirated, and cell monolayers were washed twice with PBS. Monolayers were then treated with DM containing either vehicle solution DMSO (CON) or DM + PEA (10 μM). Every 48 h, existing media was removed, monolayers washed once with PBS and fresh DM (including treatment) was introduced to monolayers up until Day 8 of differentiation. On Day 8 of differentiation, existing media was aspirated, and monolayers were washed three times with PBS. To fix the cells, paraformaldehyde (PFA 4%) solution was added and incubated at room temperature for 10 min. Thereafter, the fixative solution was removed, and monolayers were washed three times with PBS. The fixed sample can be stored for several days at 5°C. Remaining PBS was aspirated and permeabilization buffer (PBS + 0.1% Triton X‐100) was added to each well and incubated at room temperature for 15 min. Blocking buffer (10% Goat Serum in PBS, diluted 1:500) was then added and incubated at room temperature for 30 min. Monolayers were then washed once with PBS, and the primary antibody (Myosin heavy chain [MF‐20]; Mouse, 1:300, 1% BSA) (Developmental Studies Hybridoma Bank, Iowa, USA: Antibody Registry ID: AB_2147781) was added to each well, ensuring dimmed lights due to the light sensitivity of MF‐20. Plates were then wrapped in parafilm and placed in the fridge at 5°C overnight.

Following overnight incubation, the primary antibody was removed, and monolayers were washed three times with cold PBS, allowing each wash to sit for 5 min. The secondary antibody (Alexa‐Fluor 488; Goat anti‐mouse, 1:500, 1% BSA) (Thermo Scientific Inc.; Massachusetts, USA) was then added, and plates were covered with foil and left at room temperature for 60 min. Following this, the secondary antibody was removed, and monolayers were washed twice with PBS. The final step involved nuclear counterstaining with 4′,6‐diamidino‐2‐phenylindole, DAPI, a blue‐fluorescent DNA stain, diluted in H_2_O (1:100). This was added to the monolayers and incubated at room temperature for 15 min, protected from light. DAPI solution was then aspirated and PBS (200 μL) added to wells.

### 2.6 Image acquisition

A Leica DMII6000b Microscope (Leica Biosystems; Wetzlar, Germany) was used to capture fluorescently labeled monolayers as it allows for co‐visualization of MF‐20 with 4′,6‐diamidino‐2‐phenylindole (DAPI: Invitrogen D1306 purchased from Thermo Scientific Inc.; Massachusetts, USA). Images of cell monolayers were taken using the 10× objective and 0.5 magnification c‐mount fitted to the camera. Blue color channels were used as an indicator of DAPI with the wavelength measured at 358/461 nm (Fluorescent filter; EX: 340–380, DC: 400, EM: 450–490). Green color channels were used as an indicator for MF‐20, with the wavelength measured at 499/520 nm (Fluorescent filter; EX: 460–500, DC: 505, EM: 512–542). Image inspection and processing was conducted using Leica Application Suite for Windows 7 (Leica Biosystems; Wetzlar, Germany) and ImageJ 1.53a (National Institutes of Health; Maryland, USA).

### 2.7 Cell cycle analysis with propidium iodide

C2C12 myoblasts were seeded at 6 × 10^4^ cells mL^−1^ in pre‐gelatinized 6‐well plates and treated with GM containing either vehicle solution DMSO (CON) or DM + PEA (10 μM). Following 24 h treatment, existing media was aspirated, and monolayers were washed twice with PBS. To harvest cells, 200 μL of trypsin was added to each well and incubated for 5 min. Once detached, cells were pipetted into a single cell suspension in buffer (PBS + 2% FBS; PBS + 0.1% BSA). Cells were then washed with PBS and centrifuged at 3000×*g* for 5 min, this step was repeated twice, and cells resuspended at 3–6 × 10^6^ cells mL^−1^. Next, 500 μL of cells were aliquoted into a 15 mL polypropylene, V‐bottomed tube, and 5 mL of cold 70% ethanol was added dropwise while gently vortexing. Cells were then fixed for at least 1 h at 4°C on ice. After fixation, cells were washed once with PBS and centrifuged as described above again. To ensure only DNA was stained, cells were treated with Ribonuclease A to remove RNA. Fifty microlitres of RNase A solution (final concentration 0.5 μg mL^−1^) was added directly to the cell pellet. Then, 1 mL of PI solution was added, and cells vortexed and incubated overnight (or at least 4 h) at 4°C. Samples were then analyzed using a BD Accuri C6 flow cytometer (BD Biosciences, San Jose, CA, USA) equipped with four fluorescence channels (FL‐1, FL‐2, FL‐3, FL‐4). PI dye exhibits an excitation/emission maximum of 493/636 nm, which shifts to 535/617 nm upon binding, and was detected in the FL‐3 channel. Samples were acquired at a low flow rate (<400 events·s^−1^), and data collection was complete once 20,000 events had been recorded. Gating was first performed on forward and side scatter to exclude debris and aggregates, followed by fluorescence intensity gating in FL‐3 to identify positive from negative cell populations. All assays were normalized to total event counts.

### 2.8 Statistical analysis

#### 2.8.1 Dose tolerability

All statistical analysis and figures regarding dose tolerability experiments were conducted using GraphPad Prism™ for Macintosh (Version 9.3.1). All data were normally distributed, and as such were assessed with a one‐way ANOVA. Significance was assumed if α reached ≤0.05. All data are presented as mean ± standard deviation (SD).

#### 2.8.2 Myotube morphology

All statistical analysis and figures regarding myotube morphology experiments were conducted using GraphPad Prism™ for Macintosh (Version 9.3.1). Myotube number and NFI (%) data were normally distributed, and as such were assessed with an unpaired *t*‐test. Myotube area (μm^2^) data were not normally distributed, so were assessed with a Mann–Whitney *U* test. For all data, significance was assumed when *α* ≤0.05. All data are presented as mean ± standard deviation (SD).

#### 2.8.3 Cell Cyle analysis

All statistical analyses and figures regarding cell cycle progression experiments were conducted using GraphPad Prism™ for Macintosh (Version 9.3.1). All data were normally distributed, and as such were assessed with a two‐way ANOVA. Post hoc comparisons were conducted using Bonferroni's multiple comparisons test to determine differences between conditions at each cell cycle phase.

### 2.9 Bioinformatics

To visualize gene interactions and clusters of proteins that they encode with known physical interactions, STRING (string.db.org) was deployed on commonly up regulated and down regulated DEGs. Networks were exported to Cytoscape (v10.3 for Mac) to generate figures shown in results section. Protein–protein interaction (PPI) networks were constructed using known interaction data and analyzed using the Markov Clustering (MCL) algorithm to identify functionally related gene clusters. MCL was applied by simulating random walks through the network, with iterative expansion and inflation steps to enhance the separation of densely connected regions. Clustering was performed using an inflation parameter of 0.2 to control cluster granularity. Gene Ontology (GO) term enrichment was assessed for each cluster to determine functional associations. Single‐node genes with no known interactions were excluded from visualization.

Circos plots were made using RStudio v2014.12.0 using the following packages circlize v0.4.16, dplyr v1.1.4, readr v2.1.5, tidyr v1.3.1, complexheatmap v2.24.1, grid v4.5.0, and tibble v3.3.0.

**FIGURE 1:**
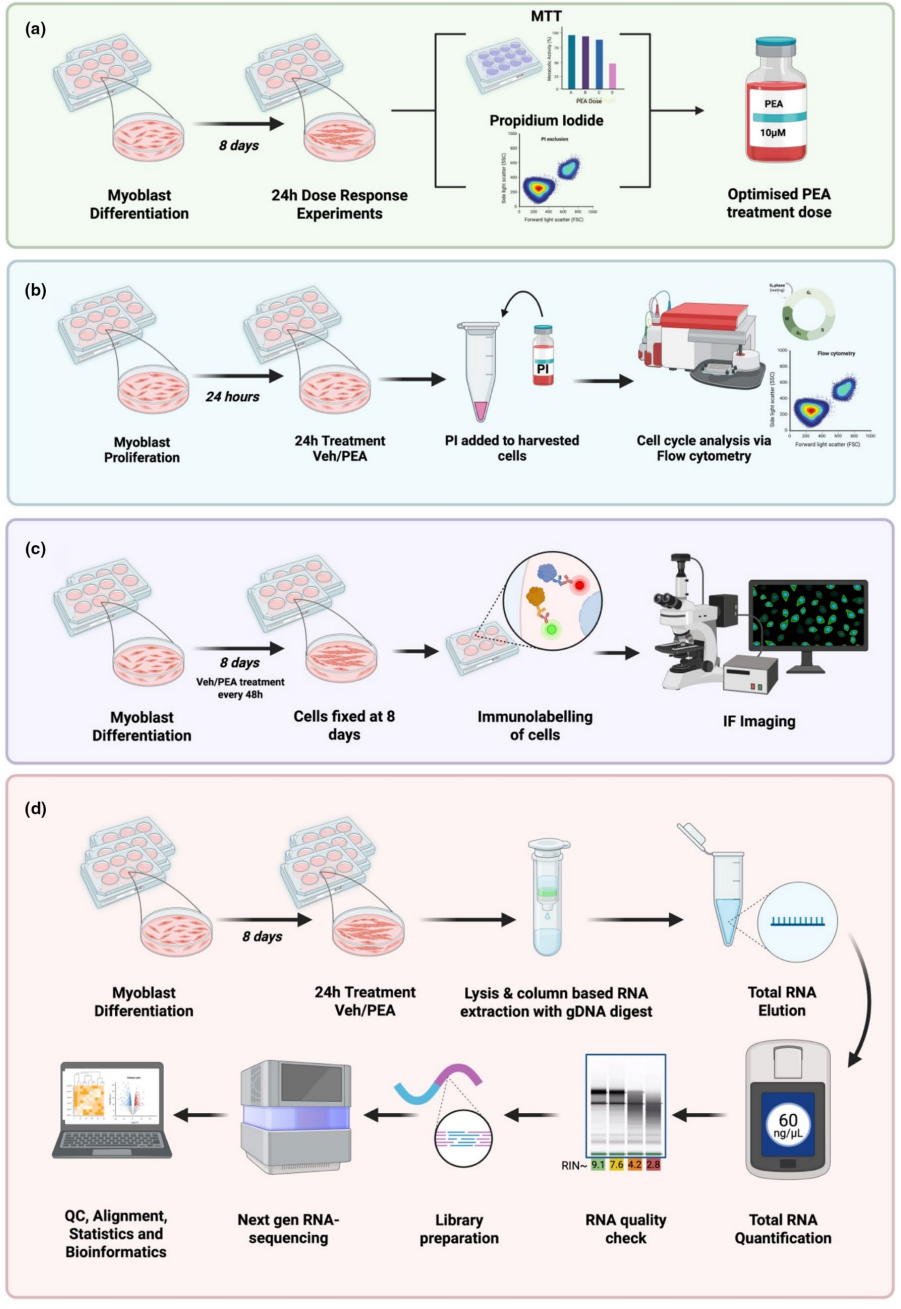
Experimental workflow for (a) the determination of dose tolerability. C2C12 myoblasts were differentiated in GM before 24 h treatment with PEA or vehicle (DMSO) control. MTT and PI exclusion assays were performed to determine the maximal tolerable dose of PEA. In workflow (b), C2C12 myoblasts were treated with PEA (10 μM) or vehicle control for 24 h. Myoblasts were then harvested and stained for DNA labelling with PI. Cell cycle phase distribution was then analyzed using flow cytometry. In workflow (c), C2C12 myotubes were treated with PEA (10 μM) or vehicle control every 48 h throughout differentiation. On day 8, myotubes were fixed, stained with DAPI and MF‐20, and imaged by immunofluorescence for morphological analysis. Image created using Biorender. Finally, in workflow (d), differentiated myotubes were exposed to PEA (10 μM) or vehicle control for 24 h before being lysed for column‐based RNA extraction. Total RNA quantity and quality were determined prior to library preparation and next‐generation sequencing. Pre‐alignment QC was performed prior to quality trimming and STAR alignment, followed by quantification to the annotation model, counts were then normalized by median ratio, prior to DESeq2 statistical analysis.

Experimental workflows are presented schematically in Figure 1.

## 3 RESULTS

### 3.1 Dose tolerability

**FIGURE 2:**
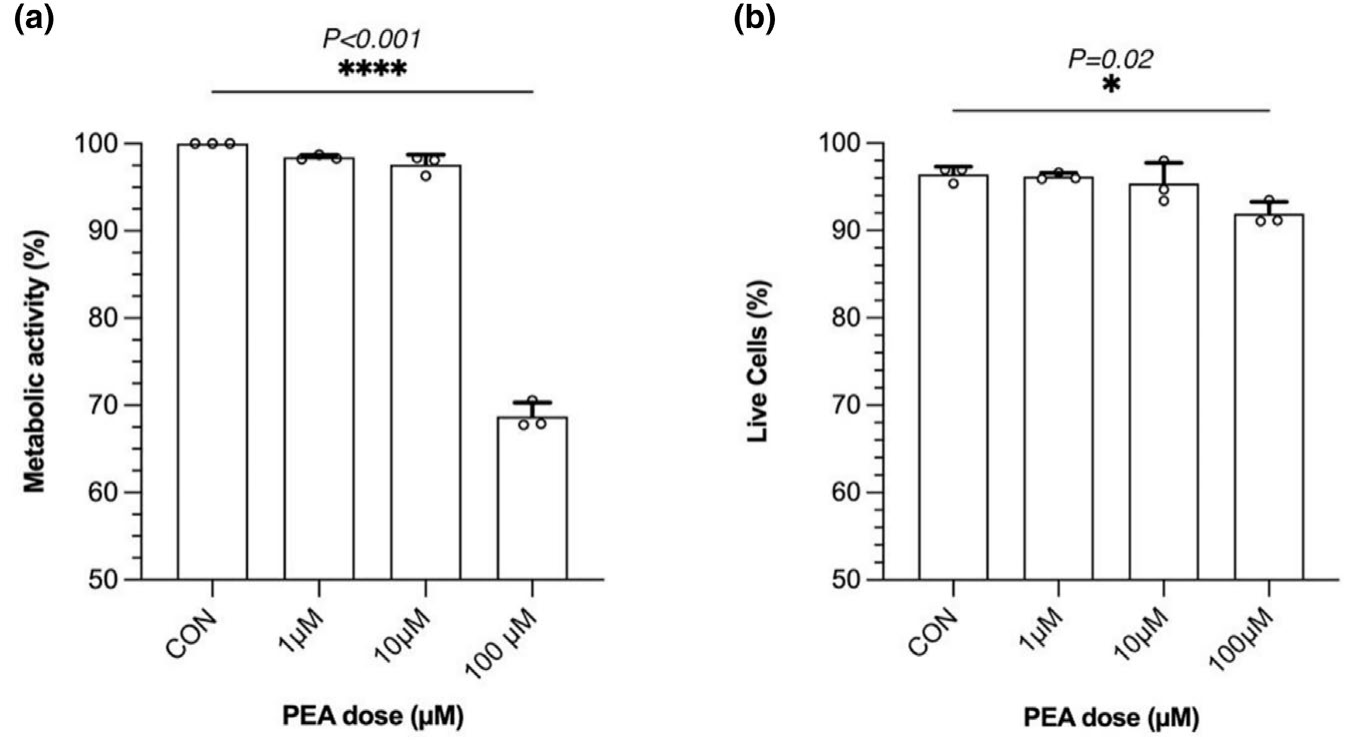
Dose tolerability experiments on terminally differentiated myotubes. Metabolic activity (a) and cell viability (b) were determined by the MTT assay and propidium iodide (PI) exclusion in PEA‐treated C2C12 myotubes over a range of concentrations (1–100 μM). Data are presented as mean ± SD from *n* = 3 biological replicates for each dose.

Following 24 h of PEA exposure, metabolic activity, as determined by the MTT assay, was significantly reduced following 100 μM compared to control (*p* < 0.0001; Figure 2a). Additionally, there was a significant reduction in the percentage of live cells, as determined by the PI assay, at 100 μM PEA compared to control (*p* = 0.02; Figure 2b). Based on these observations, we selected the maximum 10 μM PEA to evaluate its potential effects on skeletal muscle myogenesis and the transcriptome of terminally differentiated myotubes.

### 3.2 Myotube morphology

**FIGURE 3:**
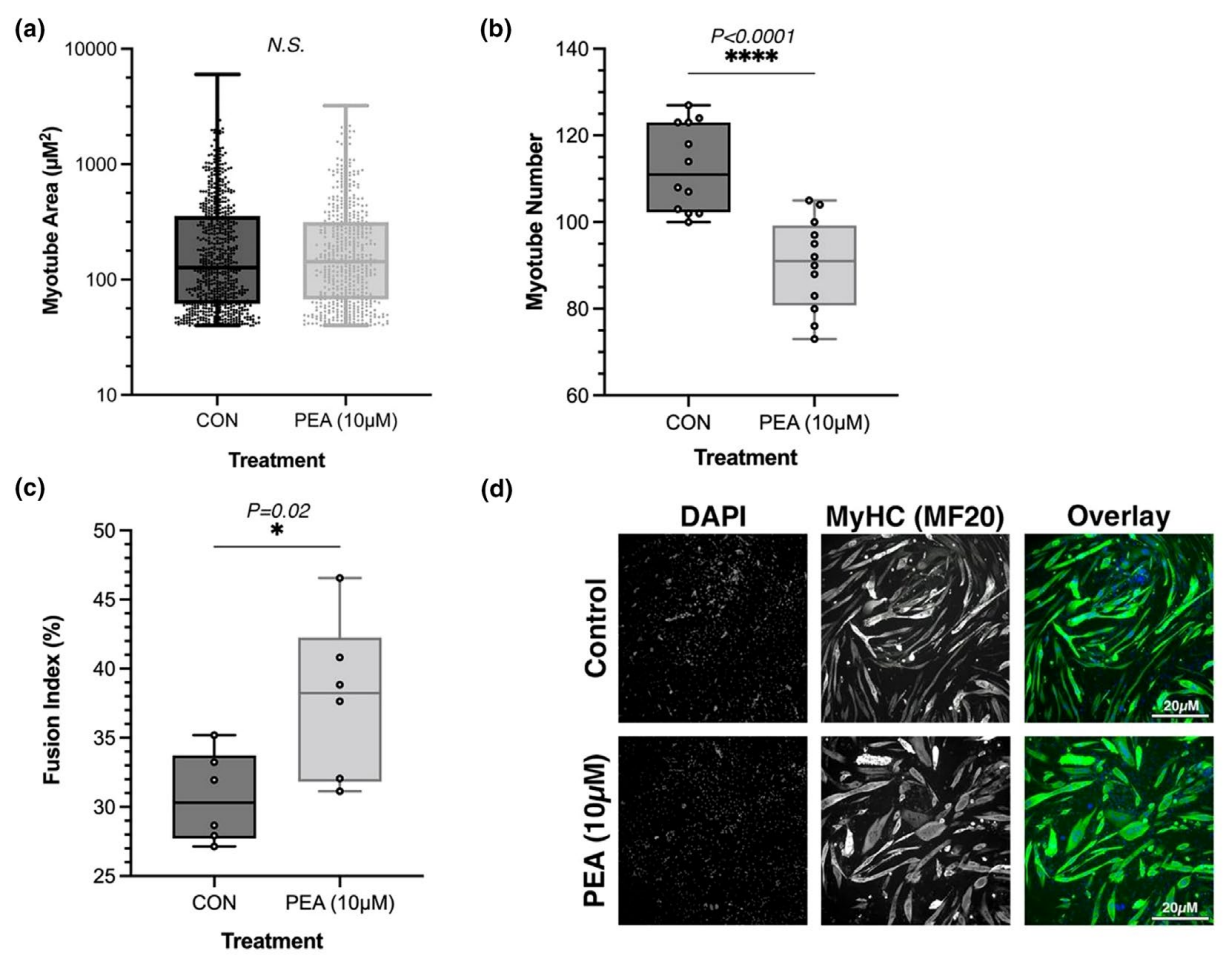
Morphological analysis of myotubes treated throughout differentiation with PEA (10 μM) or vehicle control (CON). Measurements include (a) myotube area (μm^2^), where each data point represents an individual myotube measured across six images per condition, (b) myotube number, where each data point represents the total number of myotubes per image, and (c) nuclear fusion index (NFI, %), where each data point represents the percentage of nuclei within myotubes per image. Analysis conducted using ImageJ (v1.53a). (d) Representative images of differentiated C2C12 myotubes stained for nuclei (DAPI, blue) and myosin heavy chain (MF‐20, green). One independent image is presented per condition (CON and PEA). Scale bars = 250 μM, applies to all individual channels. Quantification was performed on six randomly selected images per condition to ensure unbiased representation. Data are expressed as mean ± SD from *n* = 3 independent experimental replicates for each dose.

The morphology of C2C12 myotubes treated with either control (CON) or PEA (10 μM) throughout differentiation (every 48 h) was assessed using immunofluorescence staining for myosin heavy chain (MF‐20) and nuclear counterstaining with DAPI. Morphological analysis included quantification of myotube number, myotube area (μm^2^), and the nuclear fusion index (NFI, %). There was no significant difference in myotube area (μm^2^) between CON (296.6 ± 441.4) and PEA (284.5 ± 383) (*p* = 0.44; Figure 3a). However, there was a significant decrease in myotube number following PEA treatment (90.3 ± 10.6) compared to CON (112.6 ± 10.1) (*p* < 0.0001; Figure 3b). Nuclear fusion index (NFI, %) was significantly increased following PEA treatment (37.8 ± 5.7) compared to CON (30.7 ± 3.2) (*p* = 0.02; Figure 3c).

### 3.3 Myoblast cell cycle phase analysis

**FIGURE 4:**
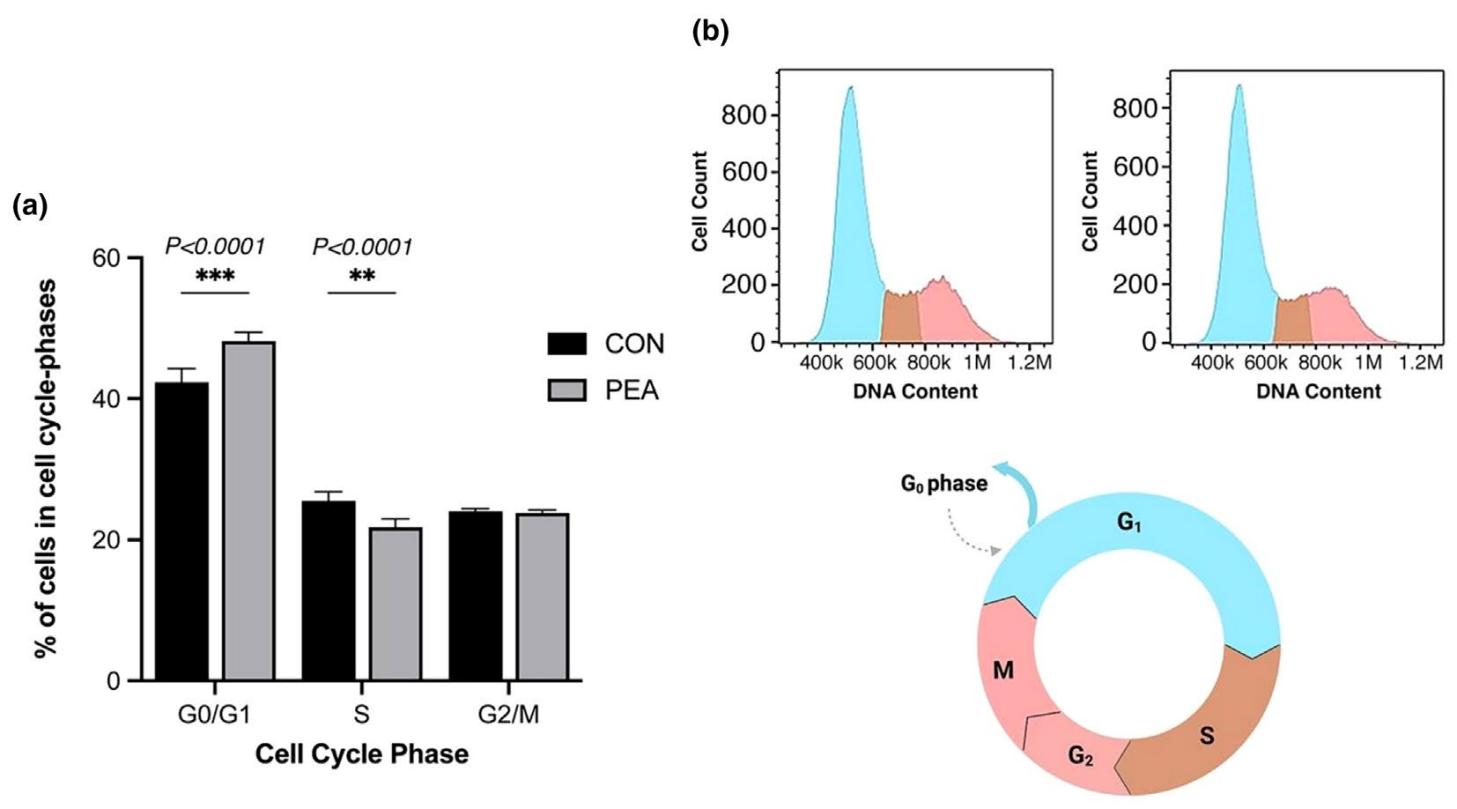
(a) Cell cycle distribution of C2C12 myoblasts following 24 h treatment with PEA (10 μM) or vehicle control (CON), assessed by flow cytometry using propidium iodide (PI) staining. Cell cycle phases determined using appropriate gates based on PI fluorescence histograms, with G₀/G₁‐phase cells identified by lower PI intensity, S‐phase cells by intermediate intensity, and G₂/M‐phase cells by higher intensity. (b) Representative PI fluorescence histogram (FL2‐H, PE channel) illustrating DNA content profiles. Peaks correspond to G₀/G₁ (light blue), S‐phase (brown), and G₂/M (red) cells. Analysis performed using FloJo software (v10). Data are presented as mean ± SD from *n* = 3 experimental replicates for each dose.

To further investigate the effects of PEA on C2C12 skeletal muscle cells, we assessed its impact on cell cycle progression using propidium iodide (PI) staining. There was a significant main effect of cell cycle phase (*p* < 0.0001), with a significant interaction between condition and phase also observed (*p* < 0.0001). Bonferroni's multiple comparisons test revealed that PEA treatment (10 μM for 24 h) led to a significant accumulation of cells harboring in the G0/G1 phase (48.2 ± 1.2%) compared to CON (42.3 ± 1.9%) (*p* = 0.0002), with a concomitant significant reduction in the proportion of cells in the S‐phase (21.7 ± 1.2%) compared to CON (25.5 ± 1.2%) (*p* = 0.008) (Figure 4).

### 3.4 Acute transcriptomic response to 24‐h of PEA exposure in C2C12 myotubes

**FIGURE 5:**
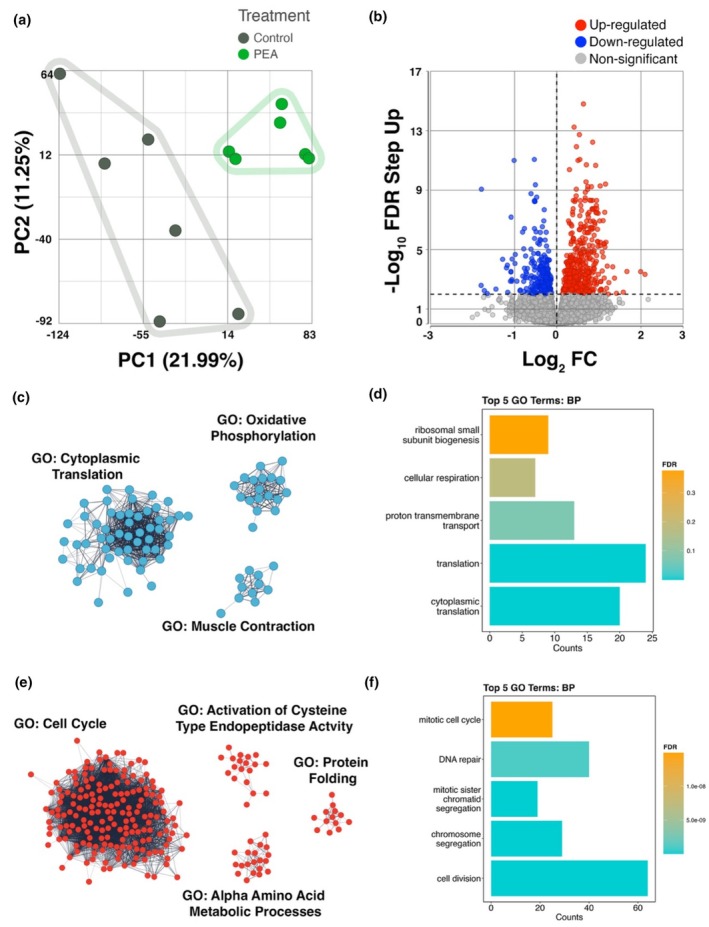
(a) Principal Component Analysis (PCA) of RNA‐seq data, illustrating the variance in gene expression between PEA‐treated and control samples (*n* = 6 per condition). Each point represents an individual sample, with clustering patterns indicating transcriptomic differences between conditions. Data are presented from *n* = 3 biological replicates for each dose. (b) Volcano plot depicting differentially expressed genes (DEGs), with −log_10_ FDR step‐up on the *y*‐axis and log_2_ fold‐change on the *x*‐axis. Gray points represent non‐significant genes, blue points indicate downregulated genes, and red points indicate upregulated genes. (c) Clusters of uniquely downregulated genes and their associated Gene Ontologies. (d) Gene Ontology (GO) biological processes for all downregulated genes. (e) Clusters of uniquely upregulated genes and their associated Gene Ontologies. (f) GO biological processes for all upregulated genes. Single‐node genes are excluded. Markov Clustering (MCL) was performed using the STRING app in Cytoscape (3.10.3).

The PCA plot in Figure 5a visualizes the multidimensional relationships among the gene expression profiles of CON and PEA treated cells. The plot reveals distinct clusters of gene expression profiles between conditions. Using *q* < 0.05 a, we found that 1952 genes were differentially expressed in PEA compared to CON. Of these genes, 1028 were significantly upregulated and 924 were significantly downregulated.

### 3.5 Transcriptional response of PEA metabolizing enzymes in skeletal muscle

**FIGURE 6:**
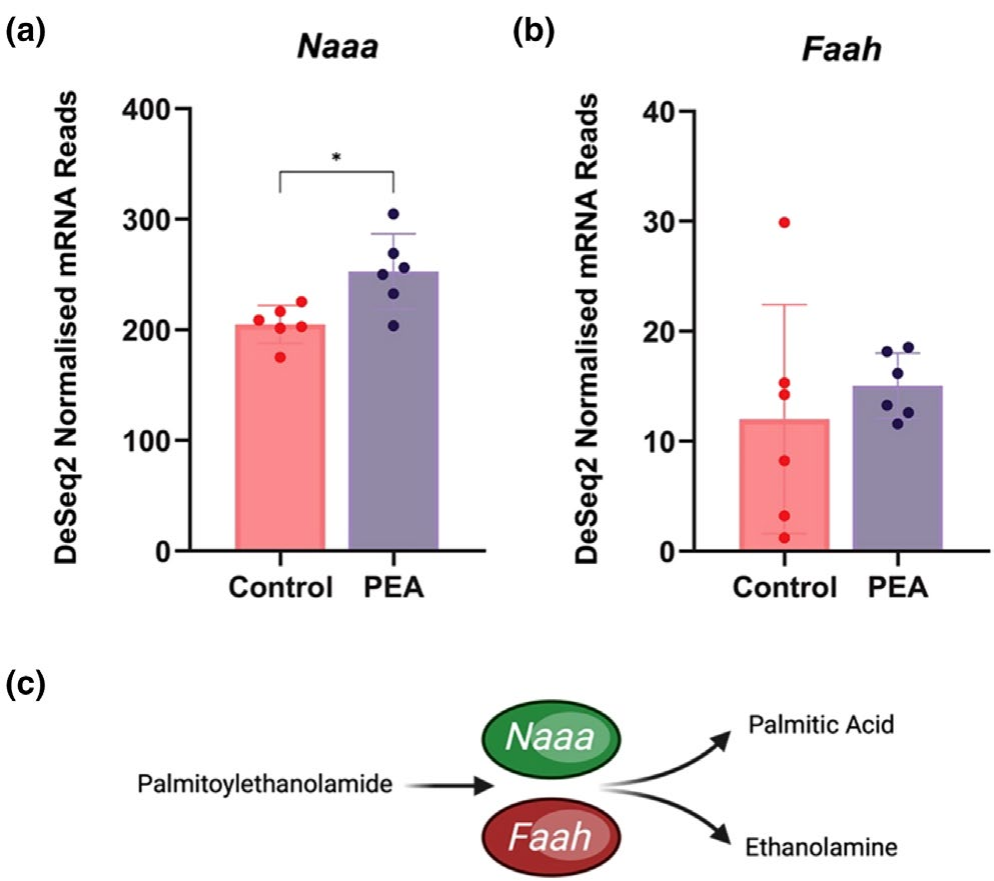
DESeq2 normalized mRNA reads for (a) N acylethanolamine acid amidase (*Naaa*) and (b) fatty acid amide hydrolase (*Faah*). (c) graphical representation of the role *Naaa* and *Faah* enzymes play in PEA metabolism.

Two enzymes are reported to be responsible for PEA hydrolysis to palmitic acid and ethanolamine; N‐acylethanolamine acid amidase (*Naaa*) and fatty acid amide hydrolase (*Faah*) (Figure 6c). Following stimulation of myotubes with 10 μM, the expression of *Naaa* was significantly (Figure 6a) upregulated vs. control, whereas *Faah* expression was unaltered (Figure 6b). Notably, *Naaa* reads were ~20‐fold higher than *Faah*, suggesting that *Naaa‐dependent* PEA metabolism may be more abundant in C2C12 myotubes.

### 3.6 PEA modulates the expression of interleukin genes, their receptors and inflammatory pathways

**FIGURE 7:**
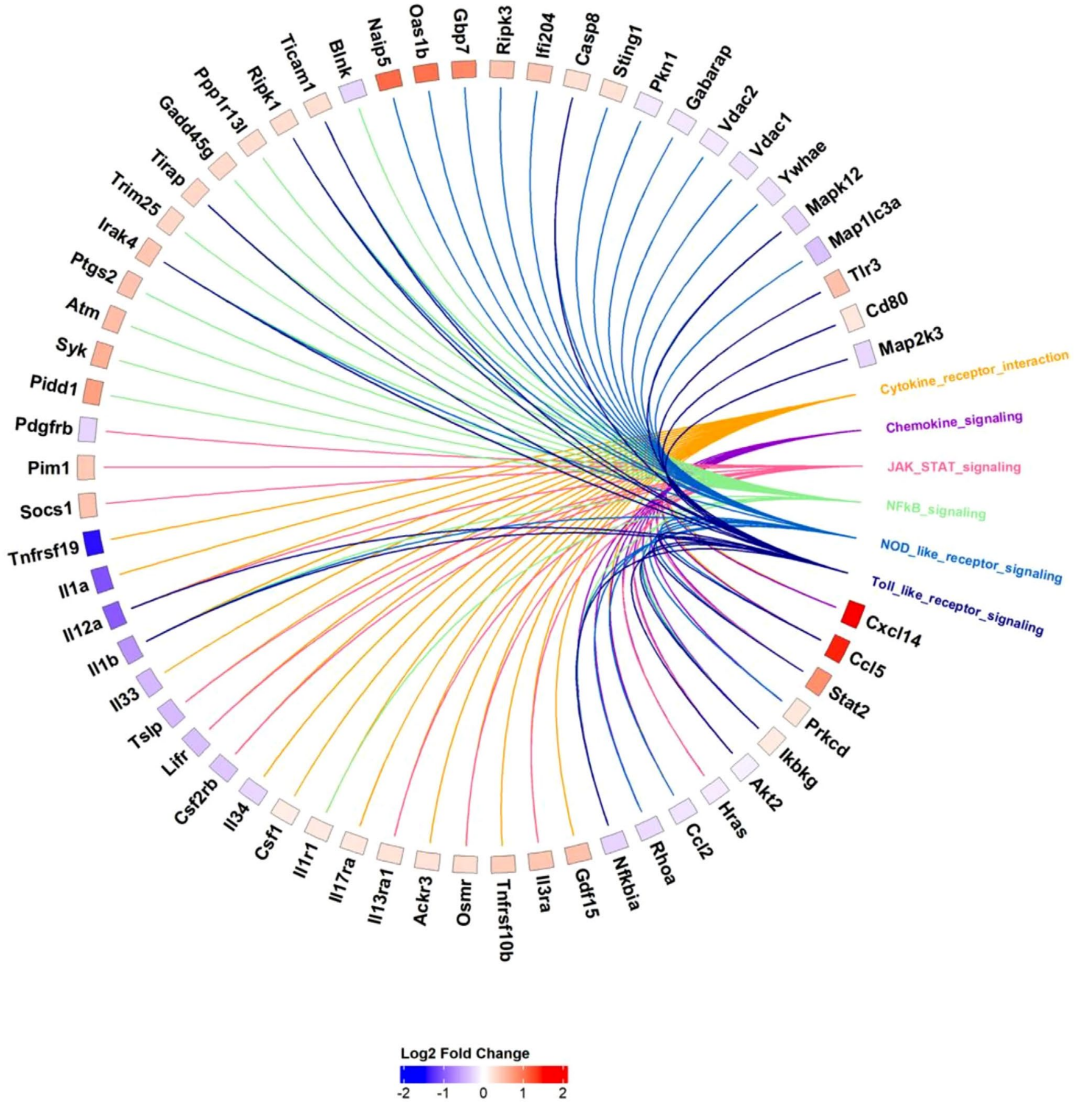
Differential gene expression in C2C12 myotubes following palmitoylethanolamide (PEA) treatment (10 μM) compared with control. Data are presented as log₂ fold change. Circos plot of differentially expressed inflammatory genes mapped to key inflammatory‐related signaling pathways. Genes are clustered into pathways and ranked by Log^2^ fold change. Genes present in multiple pathways are connected by multiple‐colored nodes.

Given the established role of palmitoylethanolamide (PEA) in modulating immune function, and emerging evidence suggesting potential actions in skeletal muscle, we investigated its role in modulating the transcriptome in C2C12 myotubes. We queried interleukins, their receptors and established inflammatory pathways (KEGG) to assess how PEA alters their expression. RNA‐seq analysis performed 24‐h following exposure to 10 μm PEA treatment revealed many genes relating to “Cytokine‐receptor‐interactions,” “Chemokine signalling,” “JAK‐STAT signalling,” “NF‐κB signaling,” “NOD‐like receptor signalling,” and “Toll‐like receptor signalling” were differentially expressed, summarized in the Circos plot which clusters genes by their associated pathway and ranks them by Log^2^ fold change (Figure 7a).

Our data supports the notion that C2C12 myotubes exposed to PEA promote an anti‐inflammatory transcriptional profile and suppress TNF signaling associated genes. Genes encoding pro‐inflammatory cytokines *Il1a, Il1b, Il33*, *Il12a*, and *Tnfrsf19* were downregulated suggesting suppression of pro‐inflammatory signaling. These cytokines are also Nuclear Factor kappa B (NF‐κB) targets suggesting reduced NF‐κB transcriptional activity, which is further supported by the downregulation of *Nfkbia*. *Socs1*, the gene encoding Suppressor of Cytokine Signaling 1, was also downregulated and is a negative regulator of the NF‐κB signaling pathway. We also observed upregulation of interferon‐related genes *Stat2*, *Oas1b*, *Gbp7*, and chemokines *Cxcl14*, *Ccl5*, suggesting immune priming while attenuating NF‐κB‐driven inflammation.

These findings and prior literature would be supportive of the hypothesis that PEA directly or indirectly activates PPAR‐α as reported in neural and immune cells. However, we found that *Ppara* was expressed at extremely low levels in C2C12 myotubes, <10 normalized counts and unaffected by PEA treatment. While we did not assess *Ppara* activity or protein abundance directly, our data suggest that PEA's effects may not be wholly through modulation of *Ppara* activity, but through *Ppara‐independent* mechanisms, potentially involving alternative immune‐modulatory pathways.

## 4 DISCUSSION

The aims of this study were to (i) determine whether palmitoylethanolamide (PEA) influences skeletal muscle myogenesis and (ii) characterize the acute transcriptional response of differentiated myotubes to PEA treatment. We observed that PEA exposure accelerated cell cycle exit and elicited a distinct transcriptomic profile, with 1952 genes differentially expressed compared with controls (1028 upregulated and 924 downregulated). Notably, expression of N‐acylethanolamine acid amidase (*Naaa*) was markedly higher and significantly upregulated, whereas fatty acid amide hydrolase (*Faah*) exhibited low expression and no significant change, suggesting *Naaa* may be the dominant enzyme for PEA hydrolysis in muscle. The transcriptional landscape also featured upregulation of cytokine receptors and apoptosis‐related genes, indicative of heightened immune responsiveness, alongside downregulation of pro‐inflammatory cytokines and NF‐κB regulators, consistent with dampened inflammatory signaling potentially via alternative pathways. Strikingly, peroxisome proliferator‐activated receptor alpha (*PPARα*), often considered the primary mediator of PEA's effects, was expressed at low levels in C2C12 myotubes and remained unchanged with treatment, challenging the view that PPARα is the dominant signaling route in muscle. Taken together, these findings not only support an inflammation‐resolving role for PEA in skeletal muscle but also provide, to our knowledge, the first comprehensive transcriptomic evidence in myotubes revealing immune‐modulatory and metabolic signatures that occur independently of canonical PPARα signaling.

Cell cycle analysis via flow cytometry revealed that 24‐h exposure to 10 μM significantly increased the proportion of cells in the G0/G1 phase, with a corresponding decrease in the S‐phase. This accumulation of cells in the G0/G1 phase is indicative of G1‐phase arrest and suggests that PEA may impede cell cycle progression. This aligns with previous studies in other cell types where PPARα activation has been shown to induce G0/G1 cell cycle arrest through downregulation of G1/S transition regulators such as cyclin D1, CDK4, and CDK2 (Gizard et al., 2005; Lien et al., 2013). These effects are specifically mediated by selective PPARα agonists, including WY‐14,643 and GW7647, indicating a direct ligand‐dependent role of PPARα in cell cycle regulation (Gizard et al., 2005; Lien et al., 2013). Such G1 arrest is a common feature of anti‐proliferative signaling and may explain the reduced number of myotubes observed following PEA treatment. Interestingly, although PPARα is typically considered the principal mediator of PEA's effects, it was expressed at low levels and remained unchanged following PEA treatment in our transcriptomic dataset. This suggests that PEA may exert similar anti‐proliferative effects via alternative signaling pathways. Supporting this, Pagano et al. (2021) demonstrated that ultra‐micronized PEA (um‐PEA) exerted anti‐proliferative effects on two different colon adenocarcinoma cell lines via PPARα and GPR55 antagonists. Collectively, these findings provide further evidence that PEA may regulate cell cycle progression, contributing to the suppression of myoblast proliferation observed in the present study and influencing subsequent myotube formation.

A novel finding in the current study relates to the PEA hydrolysing enzymes N‐acylethanolamine acid amidase (*Naaa*) and fatty acid amide hydrolase (*Faah*), which hydrolyse PEA to palmitic acid and ethanolamine. *Naaa* catalyzes the hydrolysis of PEA primarily in lysosomes at acidic pH using a cysteine‐based mechanism and exhibits high substrate selectivity for saturated N‐acylethanolamines (NAEs) such as PEA (Scalvini et al., 2020; Tsuboi et al., 2005; Ueda et al., 2010). In contrast, *Faah* catalyzes PEA hydrolysis less efficiently in the endoplasmic reticulum at neutral pH using a serine hydrolase mechanism and preferentially hydrolyses unsaturated NAEs such as anandamide (Ahn et al., 2008; Cravatt et al., 1996). Our data indicate that exposure of skeletal muscle myotubes to 10 μM PEA led to significant induction of *Naaa* gene expression versus control. In contrast, *Faah* expression was unaltered. Notably, *Naaa* reads were ~20 fold higher than *Faah* suggesting that *Naaa* dependent PEA hydrolysis may be the dominant mechanism of metabolism in muscle cells and requires further targeted experiments to confirm this.

Finally, PEA is known for its anti‐inflammatory and neuroprotective properties. Our data support this in skeletal muscle cells, as PEA‐treated muscle cells exhibited a distinct transcriptional profile characterized by the downregulation of NF‐κB target genes including *Il1a*, *Il1b*, Il12a, *Il33*, and *Tnfrsf19*. This transcriptional shift is particularly relevant as NF‐κB activity has been widely implicated in skeletal muscle wasting and impaired regeneration (Glass, 2005; Li et al., 2008). Suppression of NF‐κB target cytokines by PEA therefore suggests a potential role in promoting a pro‐regenerative and anti‐catabolic environment. Coupled with increased fusion index, these data raise the possibility that PEA modulates muscle differentiation by reducing inflammatory signaling while permitting enhanced myonuclear accretion. However, whether this translates into functional benefits such as improved regeneration following injury or attenuation of muscle atrophy in chronic disease remains to be established. Importantly, these conclusions are based on coordinated changes in NF‐κB–regulated gene expression rather than direct assessment of NF‐κB signaling activity. While the transcriptional signature observed here is consistent with reduced NF‐κB–dependent inflammatory output, we do not claim direct biochemical inhibition of the NF‐κB pathway. Direct interrogation of NF‐κB signaling dynamics, including p65 phosphorylation, IκB degradation, or nuclear translocation, would be required to confirm pathway‐level inhibition and represents an important direction for future mechanistic studies.

An important and unexpected aspect of the transcriptional response to PEA was the apparent divergence between NF‐κB–associated inflammatory signaling and interferon‐related pathways. While NF‐κB target genes were broadly downregulated, several interferon‐responsive genes and cytokine receptors were upregulated, suggesting that PEA does not induce global immunosuppression but instead promotes selective immune reprogramming. This pattern is consistent with emerging models in which resolution of inflammation involves suppression of pro‐inflammatory NF‐κB–driven cytokine networks alongside preservation or enhancement of innate immune surveillance and stress‐response pathways. Interferon signaling has been implicated in cellular differentiation, antiviral defense, and immune homeostasis, and may therefore reflect an adaptive, protective response rather than a pro‐inflammatory state. Such pathway divergence supports the concept that PEA acts as an immunomodulator rather than a simple anti‐inflammatory agent, selectively dampening deleterious inflammatory signaling while maintaining or enhancing context‐appropriate immune responsiveness.

### 4.1 Limitations

A major challenge in cell culture experiments is the translation of findings to in vivo conditions, particularly regarding the concentrations of compounds under investigation. Briskey et al. (2020) reported that following the consumption of 300 mg of PEA, peak plasma concentrations reached ~19.08 pmol·mL^−1^ at 125 min, equivalent to about 0.019 μM. To enhance absorption, they also examined Levagen+, a PEA formulation utilizing dispersion technology. Peak plasma concentrations of Levagen + were elevated to ~27.1 pmol·mL^−1^ at 105 min post‐consumption, equivalent to about 0.027 μM (Briskey et al., 2020). It is important to note that both endogenous PEA levels and exogenously administered PEA have been reported to result in low plasma concentrations due to poor absorption (Beggiato et al., 2019; Hauer et al., 2013). While plasma concentration and what the muscle is exposed to in the extracellular fluid may differ, it is important to recognize that we used a considerably higher concentration of PEA in our in vitro studies (10 μM).

While our transcriptomics experiment investigated the effects of PEA on mature myofibers, it's important to acknowledge that our in vitro model contains a subpopulation of unfused myoblasts. During differentiation, not all C2C12 myoblasts successfully fuse into multinucleated myotubes, and previous studies have documented the presence of unfused myoblasts remaining in the culture (Veliça & Bunce, 2011). These residual myoblasts could contribute to the observed expression of cell‐cycle‐related transcription factors/genes, as they may still be actively proliferating or transitioning into a differentiated state. This variability within the cell population should be considered when interpreting our results.

Messenger RNA provides a valuable molecular snapshot of the cellular response to perturbations in homeostasis and typically precedes changes in protein synthesis. However, the extent to which transcript abundance reflects alterations in peptide synthesis remains uncertain, and we cannot exclude the possibility of a “first exposure” effect, whereby an initial challenge may not reliably reproduce the transcriptomic shifts observed here.

Finally, while transcriptomic profiling provides a high‐resolution and unbiased assessment of cellular responses to PEA, we did not perform orthogonal biochemical validation of signaling pathway activity. As such, pathway‐level interpretations are based on gene expression patterns and enrichment analyses rather than direct measurements of protein phosphorylation or subcellular localisation.

### 4.2 Conclusion

Taken together, this study provides the first comprehensive transcriptomic characterization of skeletal muscle myotubes exposed to PEA, revealing coordinated immune‐modulatory, metabolic, and cell cycle–regulatory responses that occur independently of canonical PPARα gene activation. The marked induction of *Naaa* relative to *Faah* suggests a muscle‐specific bias toward lysosomal PEA hydrolysis, representing a novel aspect of PEA metabolism in this tissue. These findings expand current understanding of PEA's molecular actions beyond neural and immune systems, highlighting previously unrecognized pathways through which PEA may influence muscle inflammation, regeneration, and adaptation. To facilitate further discovery and reproducibility, all RNA‐seq data from this study are openly available via the Gene Expression Omnibus (GEO accession number GSE307376). Future work should now establish whether PEA's dual actions, restricting proliferation while enhancing fusion and dampening inflammatory signaling, translate into functional benefits for muscle growth, recovery from injury, or adaptation to exercise in vivo.

## AUTHOR CONTRIBUTIONS

Paige L. Cole performed experiments, analyzed data, created figures and wrote the manuscript. Scott H. Gillham performed experiments and analyzed data. Mark R. Viggars performed bioinformatics of RNA sequencing data and created figures. Graeme L. Close conceived the experimental design and edited manuscript drafts. Daniel J. Owens directed the project, wrote the manuscript, analyzed RNA sequencing data and created figures.

## FUNDING INFORMATION

DJO received a research grant from Gencor Pacific Ltd. for a PhD studentship for PLC.

## Supporting information


Table S1

## Data Availability

All RNA‐seq data from this study are openly available via the Gene Expression Omnibus (GEO accession number GSE307376).
