# Supplementary material for: Palmitoylethanolamide (PEA) regulates cell cycle progression and promotes an anti‐inflammatory transcriptomic signature in C2C12 skeletal muscle cells
Source: Physiol Rep. 2026 Feb 15;14(4):e70780. doi: 10.14814/phy2.70780 (PMC12907578; doi:10.14814/phy2.70780)
Supplement: Supplementary file 1 — Table S1 [file PHY2-14-e70780-s001.docx]

| Sample name | Treatment | Total reads | Total alignments | Aligned | Total unaligned | Unaligned | Total unique singleton | Unique singleton | Total unique paired | Unique paired | Total non-unique paired | Non-unique paired | Total non-unique singleton | Non-unique singleton | Coverage | Avg. coverage depth | Avg. length | Avg. quality | %GC |
| --- | --- | --- | --- | --- | --- | --- | --- | --- | --- | --- | --- | --- | --- | --- | --- | --- | --- | --- | --- |
| GC-DO-10348-C1_S9 | Control | 64,126,659 | 148,292,088 | 96.86% | 2,016,012 | 3.14% | 6 | 0% | 55,332,977 | 86.29% | 6,777,663 | 10.57% | 1 | 0% | 2.90% | 119.88 | 64.46 | 33.41 | 50.52% |
| GC-DO-10348-C2_S19 | Control | 22,713,571 | 52,619,782 | 97.00% | 682,535 | 3.00% | 1 | 0% | 19,635,835 | 86.45% | 2,395,199 | 10.55% | 1 | 0% | 2.34% | 52.69 | 64.46 | 33.42 | 50.59% |
| GC-DO-10348-C3_S21 | Control | 23,564,379 | 55,805,953 | 97.51% | 587,479 | 2.49% | 8 | 0% | 20,252,265 | 85.94% | 2,724,624 | 11.56% | 3 | 0% | 2.46% | 53.24 | 64.46 | 33.41 | 50.20% |
| GC-DO-10348-C4_S8 | Control | 19,886,054 | 46,270,354 | 97.19% | 558,430 | 2.81% | 10 | 0% | 17,190,309 | 86.44% | 2,137,305 | 10.75% | 0 | 0% | 2.18% | 49.73 | 64.46 | 33.43 | 50.39% |
| GC-DO-10348-C5_S17 | Control | 15,546,529 | 36,181,618 | 97.27% | 423,831 | 2.73% | 4 | 0% | 13,472,335 | 86.66% | 1,650,359 | 10.62% | 0 | 0% | 2.11% | 40.19 | 64.46 | 33.43 | 50.73% |
| GC-DO-10348-C6_S3 | Control | 24,091,894 | 56,408,542 | 97.02% | 717,115 | 2.98% | 2 | 0% | 20,681,513 | 85.84% | 2,693,262 | 11.18% | 2 | 0% | 2.30% | 57.53 | 64.46 | 33.42 | 50.47% |
| GC-DO-10348-P1_S22 | PEA | 23,008,571 | 53,412,677 | 96.97% | 696,750 | 3.03% | 3 | 0% | 19,839,086 | 86.22% | 2,472,731 | 10.75% | 1 | 0% | 2.36% | 53.22 | 64.46 | 33.42 | 50.46% |
| GC-DO-10348-P2_S27 | PEA | 20,274,123 | 46,544,990 | 97.31% | 545,769 | 2.69% | 4 | 0% | 17,756,195 | 87.58% | 1,972,155 | 9.73% | 0 | 0% | 2.43% | 44.90 | 64.46 | 33.45 | 51.12% |
| GC-DO-10348-P3_S4 | PEA | 22,287,816 | 51,565,237 | 96.89% | 692,756 | 3.11% | 5 | 0% | 19,240,156 | 86.33% | 2,354,898 | 10.57% | 1 | 0% | 2.34% | 51.71 | 64.46 | 33.42 | 50.40% |
| GC-DO-10348-P4_S18 | PEA | 23,650,087 | 54,604,957 | 97.14% | 676,699 | 2.86% | 1 | 0% | 20,541,739 | 86.86% | 2,431,648 | 10.28% | 0 | 0% | 2.29% | 55.97 | 64.46 | 33.42 | 50.56% |
| GC-DO-10348-P5_S2 | PEA | 23,336,888 | 53,723,675 | 97.13% | 669,875 | 2.87% | 6 | 0% | 20,296,373 | 86.97% | 2,370,633 | 10.16% | 1 | 0% | 2.29% | 55.06 | 64.46 | 33.42 | 50.67% |
| GC-DO-10348-P6_S15 | PEA | 28,064,149 | 64,881,658 | 97.30% | 757,432 | 2.70% | 6 | 0% | 24,442,616 | 87.10% | 2,864,094 | 10.21% | 1 | 0% | 2.58% | 59.10 | 64.46 | 33.42 | 50.69% |

**Supplementary Table 1.** Post alignment QA/QC report.
